# Prebiotic fibre mixtures counteract the manifestation of gut microbial dysbiosis induced by the chemotherapeutic 5-Fluorouracil (5-FU) in a validated in vitro model of the colon

**DOI:** 10.1186/s12866-024-03384-4

**Published:** 2024-06-26

**Authors:** Janine Ziemons, Lars E. Hillege, Romy Aarnoutse, Judith de Vos-Geelen, Liselot Valkenburg-van Iersel, Jasper Mastenbroek, Robin van Geel, David J. M. Barnett, Sander S. Rensen, Ardy van Helvoort, Lotte H. J. Dopheide, Guus Roeselers, John Penders, Marjolein L. Smidt, Koen Venema

**Affiliations:** 1 GROW - School for Oncology and Reproduction, Maastricht University, Maastricht, The Netherlands; 2 Department of Surgery, Maastricht University Medical Center+, Maastricht, The Netherlands; 3 Department of Internal Medicine, Division of Medical Oncology, Maastricht University Medical Center+, Maastricht, The Netherlands; 4 CARIM School for Cardiovascular Disease, Maastricht University, Maastricht, The Netherlands; 5 Department of Clinical Pharmacy and Toxicology, Maastricht University Medical Center+, Maastricht, The Netherlands; 6 Department of Medical Microbiology, Infectious Diseases, and Infection Prevention, Maastricht University Medical Center+, Maastricht, The Netherlands; 7 NUTRIM - School of Nutrition and Translational Research in Metabolism, Maastricht University, Maastricht, The Netherlands; 8 Danone Nutricia Research, Utrecht, The Netherlands; 9 Euregional Microbiome Center, Maastricht, The Netherlands; 10 Centre for Healthy Eating & Food Innovation, Maastricht University – Campus Venlo, Venlo, The Netherlands

**Keywords:** Gut microbiota, Cancer, Chemotherapy, 5-Fluorouracil, Pharmacomicrobiomics, SCFA, BCFA

## Abstract

**Background:**

5-Fluorouracil (5-FU) is used as an antineoplastic agent in distinct cancer types. Increasing evidence suggests that the gut microbiota might modulate 5-FU efficacy and toxicity, potentially affecting the patient’s prognosis. The current experimental study investigated 5-FU-induced microbiota alterations, as well as the potential of prebiotic fibre mixtures (M1-M4) to counteract these shifts.

**Methods:**

A pooled microbial consortium was derived from ten healthy donors, inoculated in an in vitro model of the colon, and treated with 5-FU, with or without prebiotic fibre mixtures for 72 h. Four different prebiotic fibre mixtures were tested: M1 containing short-chain galacto-oligosaccharides (sc GOS), long-chain fructo-oligosaccharides (lcFOS), and low viscosity pectin (lvPect), M2 consisting of arabinoxylan, beta-glucan, pectin, and resistant starch, M3 which was a mixture of scGOS and lcFOS, and M4 containing arabinoxylan, beta-glucan, pectin, resistant starch, and inulin.

**Results:**

We identified 5-FU-induced changes in gut microbiota composition, but not in microbial diversity. Administration of prebiotic fibre mixtures during 5-FU influenced gut microbiota composition and taxa abundance. Amongst others, prebiotic fibre mixtures successfully stimulated potentially beneficial bacteria (*Bifidobacterium*, *Lactobacillus, Anaerostipes, Weissella*, *Olsenella*, *Senegalimassilia*) and suppressed the growth of potentially pathogenic bacteria (*Klebsiella*, *Enterobacter*) in the presence of 5-FU. The short-chain fatty acid (SCFA) acetate increased slightly during 5-FU, but even more during 5-FU with prebiotic fibre mixtures, while propionate was lower due to 5-FU with or without prebiotic fibre mixtures, compared to control. The SCFA butyrate and valerate did not show differences among all conditions. The branched-chain fatty acids (BCFA) iso-butyrate and iso-valerate were higher in 5-FU, but lower in 5-FU + prebiotics, compared to control.

**Conclusions:**

These data suggest that prebiotic fibre mixtures represent a promising strategy to modulate 5-FU-induced microbial dysbiosis towards a more favourable microbiota, thereby possibly improving 5-FU efficacy and reducing toxicity, which should be evaluated further in clinical studies.

**Supplementary Information:**

The online version contains supplementary material available at 10.1186/s12866-024-03384-4.

## Background

5-Fluorouracil (5-FU) is an antimetabolite drug that is widely used as an antineoplastic agent in distinct cancer types, particularly in colorectal cancer (CRC) [1–3]. It can be used as single agent or in combination therapies (e.g. FOLFOX or FOLFIRI) [4].

The antineoplastic effects of 5-FU rely on intracellular ribosylation and sequential phosphorylation which yields the three active metabolites fluorodeoxyuridine monophosphate (FdUMP), fluorouridine triphosphate (FUTP), and fluorodeoxyuridine triphosphate (FdUTP), which ultimately cause DNA and RNA damage (Supplementary Figure S1) [3]. The majority of 5-FU is rapidly catabolized into inactive metabolites by the enzyme dihydropyrimidine dehydrogenase (DPD), which is mainly expressed in the liver [5, 6].

Next to direct infusion, 5-FU can be applied as oral prodrugs, for instance, capecitabine. Capecitabine is rapidly absorbed and metabolized to 5-FU through three activation steps (Supplementary Figure S1). Because the final step in the activation of capecitabine to 5-FU predominantly takes place in the tumour, rather than in normal tissue [7], the target-to-non-target concentration ratio is improved [8].

Although 5-FU-based treatments are commonly applied, there are two major disadvantages. First, only part of the patients responds to treatment [3, 9, 10]. Second, a significant proportion of patients experience severe toxicity, referring to treatment-related side effects such as hand-foot syndrome, diarrhoea, fatigue, and stomatitis [9–11], resulting in dose reductions or premature discontinuation of the therapy [12]. Several factors might contribute to inter-individual differences in response rates and the occurrence of toxicity. For example, distinct variants of human genes involved in 5-FU metabolism (e.g. of *DPYD*, the gene encoding for DPD), inter-individual differences in pharmacokinetic parameters as well as tumour characteristics have been reported to be involved [13–15].

In addition, there is increasing evidence that the gut microbiota might be another factor affecting 5-FU efficacy. LaCourse et al. recently reported that 5-FU inhibited the growth of tumour-derived *Fusobacterium nucleatum* [16], which is thought to promote chemoresistance and metastasis [17, 18]. However, *Escherichia coli* isolates were able to metabolize and deplete 5-FU concentrations in the supernatant, thereby reducing toxicity towards *F. nucleatum* and colon cancer cells [16]. Consequently, the co-occurrence of *F. nucleatum* and *E.coli* could protect *F. nucleatum* from 5-FU treatment, with a potential negative impact on prognosis. In line with this, Spanogiannopoulos et al. described that *E.coli* is able to metabolize and inactivate 5-FU by a DPD encoded within the *preTA* operon and that the presence of this operon interfered with efficacy of 5-FU treatment in mice [19]. Next to *E.coli, preTA* was also found in other genera, mainly of the phyla Pseudomonadota (Proteobacteria) and Bacillota (Firmicutes) [19]. Also, the studies from An et al., and Yuan et al. suggested that the efficacy of 5-FU-based treatment might at least partly rely on the presence of specific gut bacteria [20, 21].

Furthermore, 5-FU has been shown to inhibit the growth of several gut bacteria and to modulate gut microbiota composition [22–24]. Consequently, it might be hypothesized that 5-FU treatment could induce microbial dysbiosis and overgrowth of potentially pathogenic bacteria. In view of the various physiological functions of the gut microbiota and interactions with the immune system [25, 26], this is expected to negatively affect the patient’s metabolic and inflammatory state, as well as chemotherapy toxicity. In accordance with this, a study in mice showed that faecal microbiota transplantation (FMT) ameliorated (amongst others) diarrhoea, intestinal homeostasis, and inflammation during 5-FU-based chemotherapy [27].

To stimulate potentially beneficial bacteria and counteract the overgrowth of potentially pathogenic bacteria during 5-FU-based chemotherapy, prebiotic fibres could be used. Prebiotics were previously shown to stimulate the growth of *Bifidobacterium* and *Lactobacillus* [28]. Microbial fermentation of prebiotic fibres also leads to the formation of short-chain fatty acids (SCFA), which are known to have anti-inflammatory, anti-carcinogenic, and other health-promoting effects [29]. However, when prebiotic fibres are in short supply, gut bacteria can switch to energetically less favourable sources, such as amino acids, possibly resulting in an adverse gut milieu and the production of branched-chain fatty acids (BCFA) [30].

Previous research showed that different types of prebiotic fibres can exert distinct effects on the gut microbiota. The differing physicochemical properties of each fibre are important in this regard, especially their solubility, viscosity and fermentability, as well as their structural complexity [31, 32]. To combine the beneficial properties of different prebiotic fibres and to maximise the effect on the gut microbiota, prebiotic fibre mixtures were used in the current project.

Previous studies concerning the effects of 5-FU on gut microbiota were mostly conducted in rodent models or with isolated bacterial species [22, 23, 33]. The current study investigated the effect of 5-FU on a whole human-derived colon microbial consortium, using the TNO in vitro model of the colon (TIM-2) [34]. TIM-2 is a validated, dynamic, computer-controlled model that accurately simulates luminal conditions of the colon and can be inoculated with human-derived gut bacteria.

Using this approach, we aimed to investigate the in vitro effects of 5-FU on gut microbiota composition and levels of the microbial metabolites SCFA and BCFA. Our second aim was to examine whether prebiotic fibre mixtures could stimulate potentially beneficial bacteria in the presence of 5-FU and could therefore be used to prevent the manifestation of 5-FU-induced microbial dysbiosis. For this purpose, four different prebiotic fibre mixtures with distinct properties were used. Mix 1 (M1) contained short-chain galacto-oligosaccharides (scGOS), long-chain fructo-oligosaccharides (lcFOS), and low viscosity pectin (lvPect). Mix 2 (M2) consisted of arabinoxylan, beta-glucan, pectin, and resistant starch. Mix 3 (M3) was a mixture of scGOS and lcFOS. Mix 4 (M4) contained arabinoxylan, beta-glucan, pectin, resistant starch, and inulin.

In view of the high prevalence of suboptimal 5-FU efficacy and the experience of toxicity, more knowledge concerning the effects of 5-FU on the gut microbiota and potential strategies to counteract undesirable shifts is of great clinical importance and might significantly contribute to the optimization of 5-FU treatment.

## Methods

### Inclusion of donors

Between July 2021 and November 2021, ten healthy donors were included in Maastricht, the Netherlands. Eligible participants were postmenopausal women aged 55 years and older with no signs of breast cancer on mammography and without signs of CRC based on a recent negative immunochromatographic faecal occult blood test (iFOBT) home test which was applied for routine CRC screening [35]. The exclusion criteria included cancer in history, inflammatory bowel disease, mammography older than 12 months, iFOBT home test older than 12 months, and therapeutic antibiotics use within three months before faecal sampling.

### Sample collection and preprocessing

Each participant collected an anaerobic faecal sample at home, using a collection kit, which contained gloves, a plastic sampling box, the faecal collection device FecesCatcher (*Tag Hemi, the Netherlands*) as well as a plastic bag for storage of the box. All participants also received an AnaeroGen™ anaerobic gas-generating sachet (*Thermo Scientific, USA*) to keep the samples anaerobically. After collection, faecal samples were cooled and immediately transported to Maastricht University Medical Center (MUMC+) for preprocessing in the BACTRON300 anaerobic work cabinet (*Sheldon Manufacturing, Inc., USA*). The faecal sample was weighted and mixed 1:1 with a dialysate solution containing magnesium sulphate solution (50 g/L MgSO_4_), calcium chloride solution (45 g/L CaCl_2_•2H_2_O), cysteine solution (20 g/L), dial solution (25 g/L K_2_HPO_4_•3H_2_O, 45 g/L NaCl, 0.05 g/L FeSO_4_•7H2O, 1.5 g/L ox-bile, water) and sterile water [34]. Subsequently, the sample-dialysate mix was homogenized with glycerol (final concentration of 15% in the homogenate). The faecal homogenate was sieved, snap-frozen in liquid nitrogen, and stored at -80 °C for the short term (a few weeks) and − 196 °C for the long term.

In addition, each participant filled in a questionnaire concerning general medical characteristics among which were weight, length, history of abdominal surgery, smoking, alcohol usage, diabetes, medication use, pro- and prebiotic use, reproductive history, dietary habits, and questions on general performance, and wellbeing.

### TIM-2 experiments

#### TIM-2 model

The TIM-2 model has been previously described and used in earlier studies [34]. A schematic representation of the system can be found in Figure S2. The model consists of four interconnected glass compartments with flexible membranes inside, which mimic peristaltic movements. During the experiments, physiological colonic conditions were simulated by maintaining a stable temperature of 37 °C and pH of 5.8 or higher by automatic titration with 2 M NaOH. The anaerobic environment was maintained through continuous flushing of the unit with nitrogen gas. The dialysis system removed bacterial metabolites from the lumen, which would otherwise inhibit gut bacteria. Total volume was kept constant at approximately 120mL.

#### Test products

For this study, four different prebiotic fibre mixtures (M1, M2, M3, M4) were used (Table 1).

M1 and M3 provide predominantly soluble, easily fermentable prebiotics with low structural complexity, and in M1 additionally a more complex fibre (lvPect) [32]. M2 and M4 both provide a variety of naturally occurring and structurally more complex gluten-free dietary fibres, with the addition of the well-recognized prebiotic fibre inulin in M4 [31, 32]. These mixtures contain soluble and insoluble fibres, intended to stimulate a diverse and resilient microbiome across the large intestine [36].

Prebiotic fibre mixtures were suspended in the standard ileal efflux medium (SIEM) which was prepared using starch, pectin, xylan, arabinogalactan, amylopectin, TBCO (Tween 80, casein, bactopeptone, and ox bile), magnesium sulphate solution, cysteine solution, vitamins, salts, and antifoam B emulsion, as described by Cuevas-Tena et al. [37]. The fibre fraction of SIEM reflected the average non-digestible carbohydrates consumed in a regular western diet. SIEM ± prebiotics was continuously (2.5mL/h) applied during the day (7.5 g of prebiotic / day).

In addition, the gut microbiota was treated twice a day (1x morning, 1x afternoon) with 26.4 mg 5-FU (Merck, USA*)*, dissolved in 10mL of dialysate solution. The concentration was chosen based on the assumption that 2.64% of an orally administered capecitabine dose is excreted via the faeces, either as capecitabine or as its downstream metabolites (see Supplementary Table S1 for more details about the calculation) [8]. In a pilot experiment, we tested 100% of the calculated dose (= 2 × 52.8 mg) and 50% of the calculated dose (= 2 × 26.4 mg). Because both doses induced comparable shifts in gut microbiota composition (Supplementary Figure S3), we chose to use the 50% dose for further experiments. This accounts for the fact that 5-FU normally reaches the colon gradually, compared to the 5-FU shots applied in this experimental setting.

**Table 1:** Composition of prebiotic fiber mixtures used for the current study

	Compound 1	Compound 2	Compound 3	Compound 4	Compound 5	Ratio
M1	scGOS	lcFOS	lvPect	-	-	9:1:2
M2	arabinoxylan	beta-glucan	lvPect	resistant starch	-	5:2:2:1
M3	scGOS	lcFOS	-	-	-	9:1
M4	arabinoxylan	beta-glucan	lvPect	resistant starch	inulin	5:2:2:1:3

scGOS: short-chain galacto-oligosaccharide; Vivinal GOS Powder, Royal Friesland Campina, derived from lactose

lcFOS: long-chain fructo-oligosaccharides; Orafti HP, Beneo, derived from chicory root

lvPect: low viscosity pectin; SF 50 LV, Herbstreith & Fox, derived from apple

arabinoxylan: corn fiber, Clonbio, derived from corn

beta-glucan: PromOat gluten-free, Lantmännen, derived from oat

resistant starch: Novelose 330, Ingredion, derived from corn

inulin: Orafti ST, Beneo, derived from chicory root

#### Procedure

**Fig. 1:**
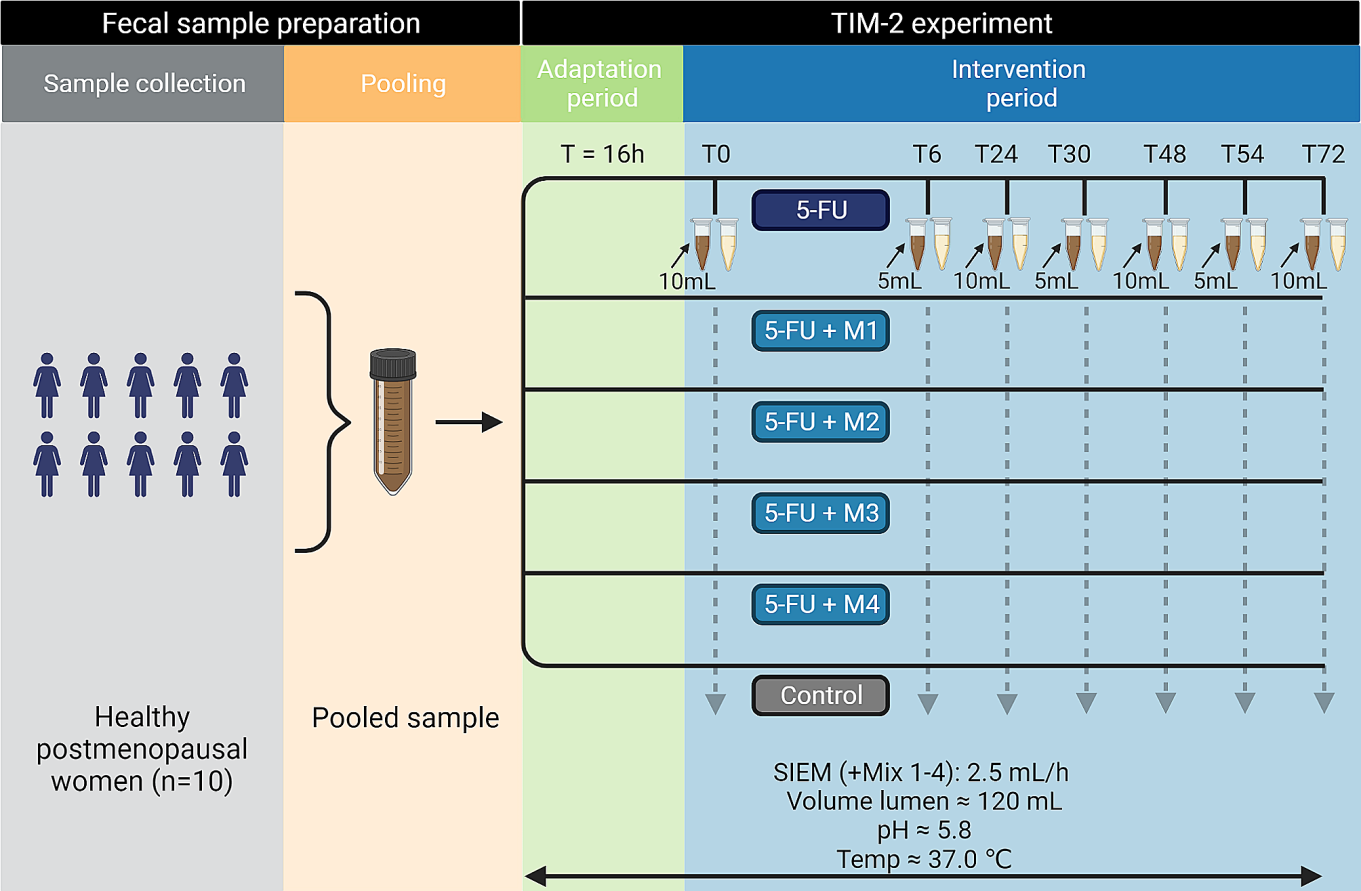
TIM-2 experimental design. Ten healthy postmenopausal women collected faecal samples and stored them anaerobically. These samples were pooled into one standardized microbiota. This pooled faecal material was inoculated into each of the TIM-2 units and fed with a standard ileal efflux medium (SIEM) for 16 h. After this adaptation period, 5-FU was introduced to the TIM-2 units at T0, T6, T24, T30, T48, T54, and T72, except for the control units. Before 5-FU injection, luminal samples and dial output samples were collected for further analysis. At T0, T24, T48 and T72 10mL lumen was retracted, whilst at T6, T30 and T54 only 5mL was retracted. Throughout the intervention period, the model was continuously fed with SIEM or SIEM + prebiotic fibre mixtures 1–4 (M1-4) respectively. Each condition was tested in duplicate. Luminal volume, pH, and temperature were kept constant during the entire course of the adaptation and intervention periods. Created with BioRender.com

An overview of the experimental procedure is provided in Fig. 1. The pre-processed faecal homogenates were thawed at 37 °C for one hour under anaerobic conditions and were then pooled together. This pooling enabled us to use the same inoculum composition for multiple experimental runs and has been previously shown to be a suitable technique for fermentation experiments [38].

The following procedure was based on the previously published protocol [34]. Before introduction into the TIM-2 units, the pooled faecal material was mixed 1:1 with a dialysate solution containing the same components as mentioned in the preprocessing. Subsequently, each TIM-2 unit was inoculated with 60mL of the homogenate. To adapt to the system, the microbiota was first fed with SIEM for 16 h. After the adaptation period, the intervention was started and maintained for 72 h. At the start of the intervention, baseline samples (T0) from the lumen, as well as from the effluent of the dialysis system (dial), were collected. After this, the first 5-FU injections and prebiotic fibre mixtures were added to the test conditions. Further sampling occurred after 6-, 24-, 30-, 48-, 54-, and 72 h. Each condition was tested in duplicate, in two independent units.

### Analysis of gut microbiota composition, diversity, and bacterial abundances

DNA from 1mL of lumen samples (T0, T24, T48, T72) was isolated with the QIAamp Fast DNA Stool mini kit (Qiagen, the Netherlands), adapted to increase the DNA yield as described by Knudsen et al. [39].

Subsequently, sequencing of the V3-V4 regions of the 16 S rRNA gene was performed according to the manufacturer’s instructions (Illumina, USA). PCR primers were based on Klindworth et al. [40], the complete primer sequences can be found in Table S2. AMPure XP beads were used to purify the 16 S rRNA gene V3-V4 amplicon. The Nextera XT Index kit was then used to attach dual indices and Illumina-sequencing adapters in a second PCR reaction. Subsequently, AMPure XP beads were used again to purify the library before quantification. Afterward, samples were equimolar pooled and sequenced with the Illumina MiSeq sequencing system and the 2 × 300 bp paired-end protocol [34].

### Analysis of SCFA and BCFA concentrations

Lumen samples (T0, T6, T24, T30, T48, T54, T72) were diluted 1:4 with ice-cold phosphate-buffered saline (PBS) and centrifuged at 15,000x g and 4 °C for three minutes. From each sample, 200μL supernatant was transferred to the corresponding well in a 96-well V-Bottom plate (Falcon, Corning Incorporated, USA). From the dial samples (T0, T6, T24, T30, T48, T54, T72), 200μL were directly transferred to the plate. The plate was covered with aluminium foil and kept on ice during the entire procedure. Subsequently, concentrations of SCFA (acetate, propionate, butyrate, valerate) and BCFA (iso-butyrate, iso-valerate) were analysed as previously described, using a Shimadzu GC2025 gas chromatograph (*Shimadzu Corporation, Kyoto, Japan*) with a flame ionization detector (split mode) [41]. The sample (0.5μL) was injected at 80 °C into a Stabilwax column (15 m × 0.53 mm, film thickness 1.00 μm; *Restek Co., Bellafonte, PA*) using H2 as the carrier gas (20.7 kPa). Prior to analysis, new columns were conditioned overnight at 200 °C. The oven temperature was programmed to increase to 160 °C first and afterwards to 220 °C at rates of 16 °C/min and 20 °C/min, respectively, and then held at 220 °C for 1.5 min. The injector and detector temperatures were both set at 200 °C. To prevent column memory effects, 0.5μL of 1% formic acid was injected after every ten samples, followed by the injection of a 0.5mL standard mix (1.77mM acetate, 1.15mM propionate, 0.72mM butyrate, 0.72mM iso-butyrate, 0.62mM valerate, and 0.62mM isovalerate; Sigma Aldrich, USA). SCFA and BCFA concentrations were determined using 2-ethylbutyric acid as standard.

### Statistical and bioinformatic analysis

#### Analysis of data from 16 S rRNA amplicon sequencing

Preprocessing was performed with an in-house pipeline, incorporating the following steps:

primer removal using cutadapt [42], read quality filtering, denoising, and chimera removal using DADA2 [43]; taxonomic classification using SILVA database release 138.1, and contaminant filtering using decontam with method = “either” [44].

After preprocessing, 1,452 Amplicon Sequence Variants (ASVs) were identified. ASVs with less than 55 reads (0.001% of total reads) and ASVs present in less than two samples (3% prevalence) were removed using an online application (https://giangle.shinyapps.io/phyloFilter/). 634 ASVs (99.7% of the data) belonging to 115 unique genera, were maintained for downstream analysis. In addition to the removal of rare ASVs, one sample (T72, 5FU + M4) was excluded for further analysis because of very low reads (< 15,000). In the remaining samples, the median number of reads was 62,814 (range: 15,940–491,462).

Downstream statistical analysis was performed using R (4.1.3) [45]. Due to the recent change of the phyla names [46], former taxonomic names are given between brackets in the text, if applicable. The R packages, phyloseq [47], vegan [48], microbiome [49], dplyr [50], ggplot2 [51], and microViz [52] were used for ordination and visualization of taxonomic composition. For visualization based on relative abundances, taxa present in less than 10% of the samples were filtered out. Composition plots were drawn by means of the *comp_barplot* function from the microViz package. Relative abundances of taxa of interest on phylum and genus level were calculated using the microbiome package and visualized with ggplot2. For unconstrained ordination, Principal Component Analysis (PCA) was performed using the *ord_plot* function from microViz based on centered log-ratio (CLR) transformed data (i.e., Aitchison distance). For PCA, taxa present in less than 5% of the samples were filtered out.

The *taxatree_models* function from the microViz package was used to perform linear regression using log2-transformed relative abundances. Taxa which were present in less 10% of the samples were filtered out. *P*-values were adjusted for multiple testing using False Discovery Rate (FDR) correction according to the method of Benjamini and Hochberg [53]. Subsequently, the results from linear regression were visually summarised in taxonomic association trees with the *taxatree_plots* function.

Temporal stability of the microbial community was expressed as genus-level Bray Curtis distances between T0/T24, T0/T48, and T0/T72 within the same run, calculated using the *dist_calc* function from microViz.

Microbial α-diversity (Shannon index) and observed species richness were calculated on genus level with the microViz package and visualized with ggplot2. The ANOVA-type statistic (ATS) from the nparLD package was used to test if α-diversity indices evolved differently over time in the conditions of interest [54]. Results from these nonparametric marginal models were confirmed by means of repeated measures ANOVA using the rstatix package [55]. For the 5-FU + M4 condition, one of the duplicates was removed for this analysis because of the missing sample at T72.

#### Statistical analysis of SCFA/BCFA data

Measured concentrations in lumen samples were multiplied by four to correct for the 1:4 diluted solution prepared during the preprocessing. Subsequently, concentrations in the lumen and dial samples were used to calculate cumulative SCFA and BCFA concentrations in the TIM-2 system during the intervention period. Concentrations were artificially set to zero at T0. The calculation accounts for the fact that a certain volume of lumen was removed from the system per time point for sampling. In the case of non-detectable concentrations, the last calculated concentration was used for visualization purposes. Longitudinal changes of cumulative SCFA and BCFA levels were visualized by means of ggplot2 [51]. The nparLD package was used to fit nonparametric marginal models, and calculate the ATS, to investigate whether 5-FU, controls, and prebiotic fibre mixture conditions evolved differently over time [54]. Results from nonparametric marginal models were confirmed by means of repeated measures ANOVA using the rstatix package [55]. In addition, we used the Kruskal Wallis test in order to test for differences between the conditions at the end of the intervention (T72).

## Results

### Participant characteristics

In total, faecal samples of ten postmenopausal women, were included and pooled. The mean age was 65 years (range: 56–72 years). The mean BMI was 22.43 kg/m^2^. None of the participants followed a special diet (e.g. vegetarian, vegan, low carb, high protein) or reported the use of antibiotics, prebiotics, or probiotics within six months prior to inclusion. Further participant characteristics are summarized in Supplementary Table S3.

### Administration of 5-FU-induced shifts in gut microbiota composition

**Fig. 2:**
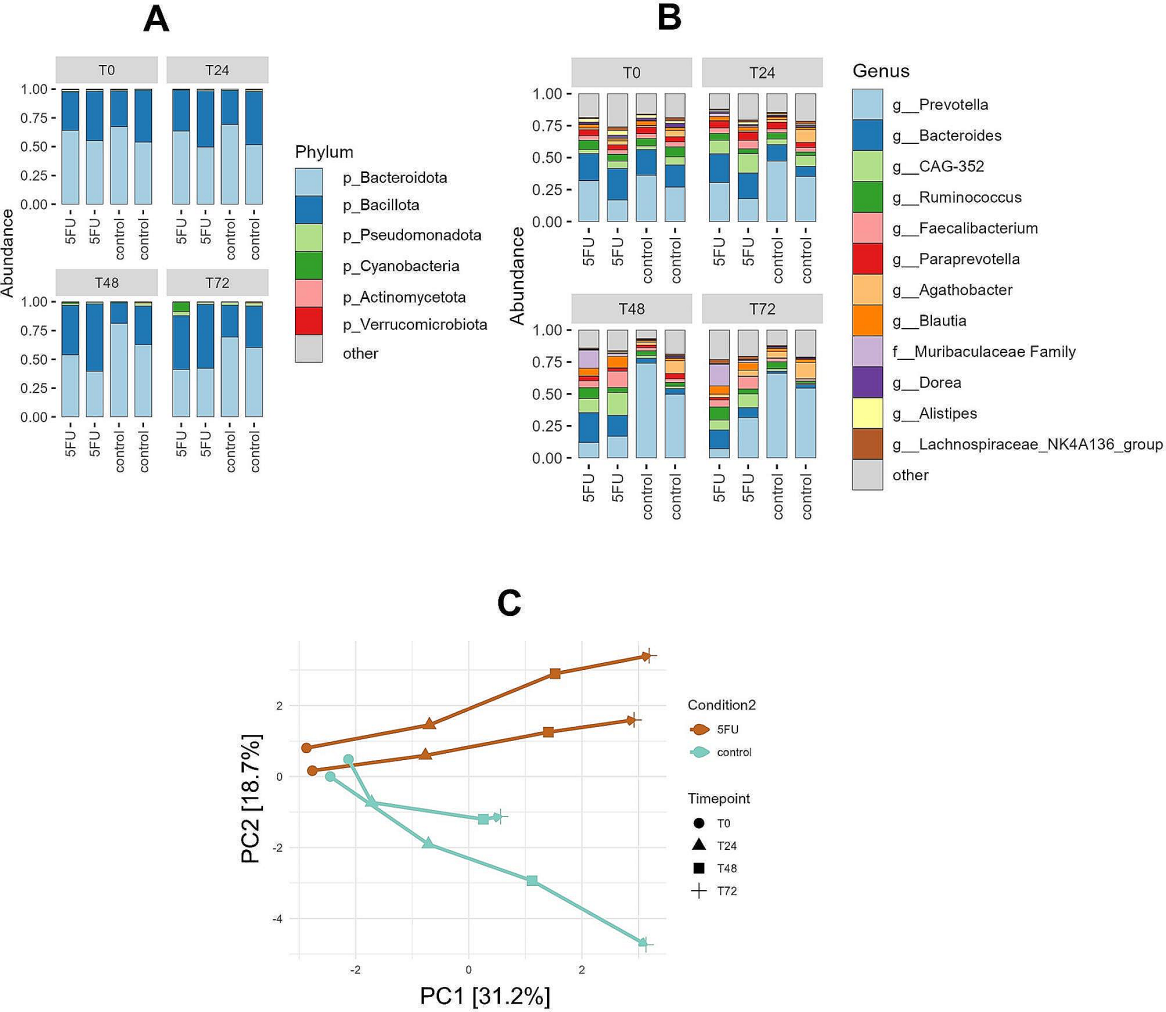
5-FU-induced shifts in overall gut microbiota composition. **A**: Changes in relative abundances of the six most common phyla at different time points during the intervention. Taxa which were present in less than 2 of the 16 samples (minimum prevalence of 10%) were filtered out. All conditions were tested in duplicate, and individual experiments are shown. **B**: Changes in relative abundances of the 12 most common genera at different time points during the intervention. Taxa which were present in less than 2 of the 16 samples (minimum prevalence of 10%) were filtered out. All conditions were tested in duplicate, and individual experiments are shown. **C**: Ordination plot derived from unconstrained PCA, showing shifts in the overall composition of the microbial community at genus level. Data were transformed using clr transformation. Arrows (running from T0 to T72) indicate individual runs over time

The administration of 5-FU induced shifts in overall gut microbiota composition, on phylum as well as genus level (Fig. 2A-C). While baseline samples clustered together, differences in gut microbiota composition between the 5-FU and control conditions became apparent during the intervention period (Fig. 2C). More specifically, we observed a shift in the Bacillota/Bacteroidota (Firmicutes/Bacteroidetes) ratio, due to a relative increase of Bacillota (Firmicutes) accompanied by a relative decrease of Bacteroidota (Bacteroidetes) in the 5-FU condition (Fig. 2A and Supplementary Figure S4). While the relative abundance of the 12 most common genera was comparable between all four samples at baseline, some genera behaved differently in the 5-FU condition during the intervention period (Fig. 2B and Supplementary Figure S5). The relative abundance of *Prevotella* increased in the control group, which was not the case for the 5-FU condition. *Bacteroides* decreased in both conditions but remained at higher levels during the 5-FU treatment. *CAG-352* (a genus from the Ruminococcaceae family), *Faecalibacterium*, and *Blautia* were relatively stable in the control condition but increased due to the 5-FU treatment.

**Fig. 3:**
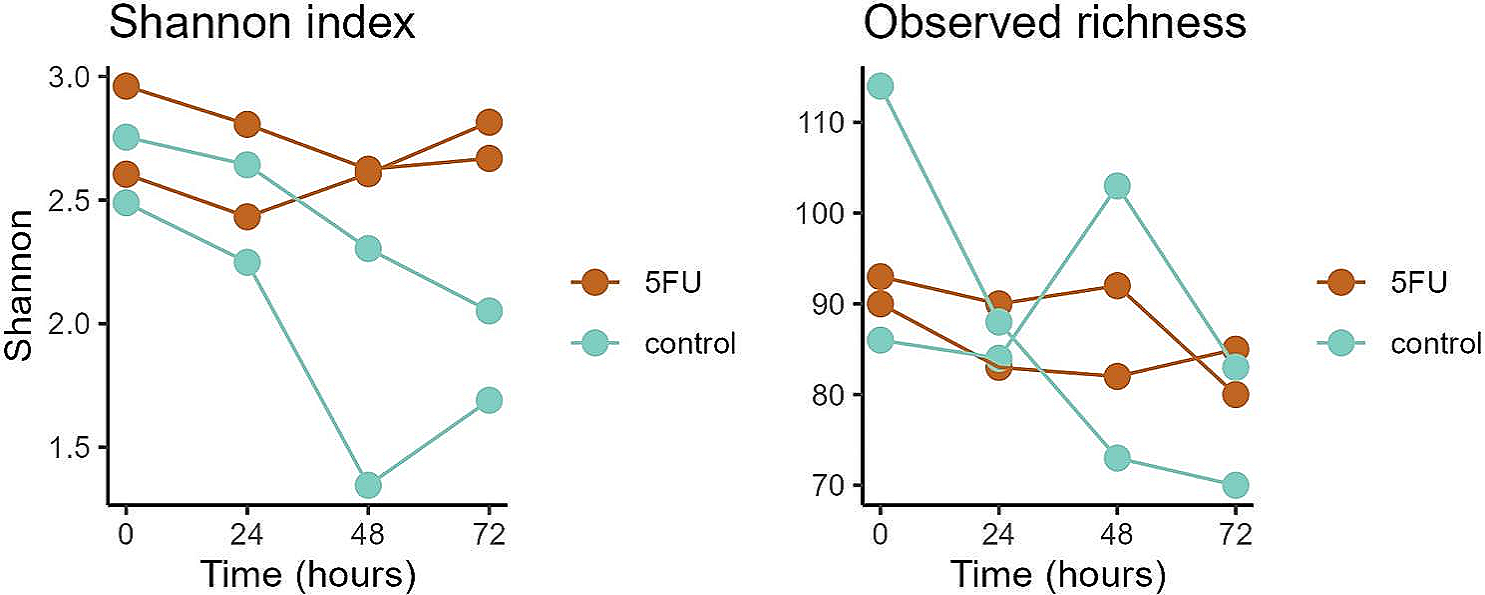
Effects of 5-FU treatment on microbial diversity (Shannon index) and observed species richness. Individual runs are plotted over time, both diversity indices were calculated on genus level

According to nonparametric marginal models, microbial α-diversity, expressed as the Shannon index (ATS: *p* = 0.167), as well as species richness (ATS: *p* = 0.939) showed no significantly different development over time (Fig. 3). These results were confirmed by repeated measures ANOVA, although the Shannon index showed a trend towards significance (Shannon index: *p* = 0.052, species richness: *p* = 0.352, Fig. 3).

### Prebiotic fibre mixtures stimulated potentially beneficial bacteria also in the presence of 5-FU

**Fig. 4:**
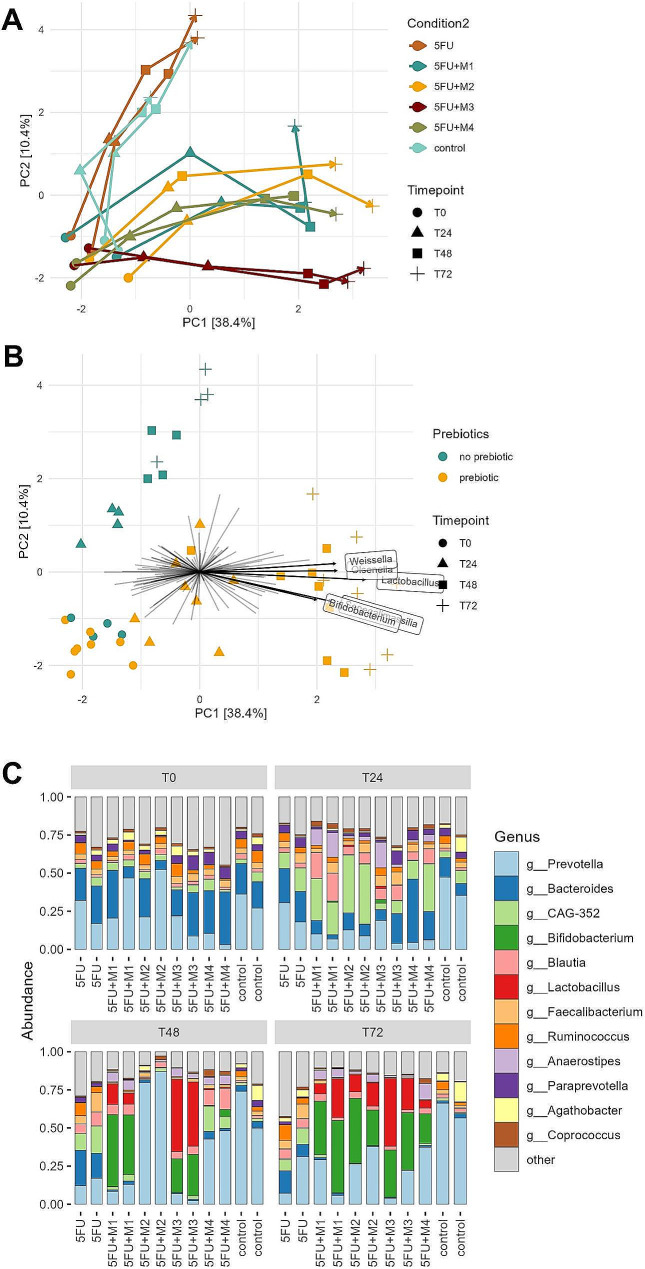
Prebiotic fibre mixtures-induced changes in overall gut microbiota composition. **A**: Ordination plot derived from unconstrained PCA based on clr transformed data, showing longitudinal shifts in overall gut microbiota composition at genus level. Taxa which were present in less than 3 of the 47 samples (minimum prevalence of 5%) were filtered out. Colours represent the different conditions, and arrows (running from T0 to T72) indicate individual runs over time. **B**: Ordination plot derived from unconstrained PCA based on clr transformed data, showing differences in longitudinal microbiota shifts between conditions with prebiotic fibre mixtures (5-FU + M1, 5-FU + M2, 5-FU + M3, 5-FU + M4) and without prebiotic fibre mixtures (5-FU, control). Taxa which were present in less than 3 of the 47 samples (minimum prevalence of 5%) were filtered out. Vectors indicate the top five taxa which contributed most to the observed variation between conditions with and without prebiotic fibre mixtures along PC1. **C**: Prebiotic fibre mixtures-induced changes in relative abundances of the 12 most common genera at different time points during the intervention. Taxa which were present in less than 5 of the 47 samples (minimum prevalence of 10%) were filtered out. All conditions were tested in duplicate and individual experiments are shown

All four prebiotic fibre mixtures induced changes in gut microbiota composition (Fig. 4A-C and Supplementary Figure S6). Baseline samples clustered together, but during the intervention period, gut microbiota composition evolved differently between the different test conditions (Fig. 4A and B). Among the 12 most common genera, the prebiotic-induced effect was mainly characterized by a relative increase in *Bifidobacterium*, *Lactobacillus*, and *Anaerostipes* (Fig. 4C). While the more soluble and less complex mixtures M1 and M3 already had a strong prebiotic effect at T48, the prebiotic effects of the more diverse and complex fibres in M2 and M4 were only visible at T72 (Fig. 4C). PCA indicated that the genera *Weissella*, *Olsenella*, *Lactobacillus*, *Senegalimassilia*, and *Bifidobacterium* contributed most to the observed difference between the conditions with (5-FU + M1, 5-FU + M2, 5-FU + M3, 5-FU + M4) and without (5-FU, control) prebiotic fibre mixtures along PC1 (Fig. 4B). Along PC2, the genera *Klebsiella*, *Enterobacter*, and *Tyzzerella* contributed the most (Supplementary Figure S7).

**Fig. 5:**
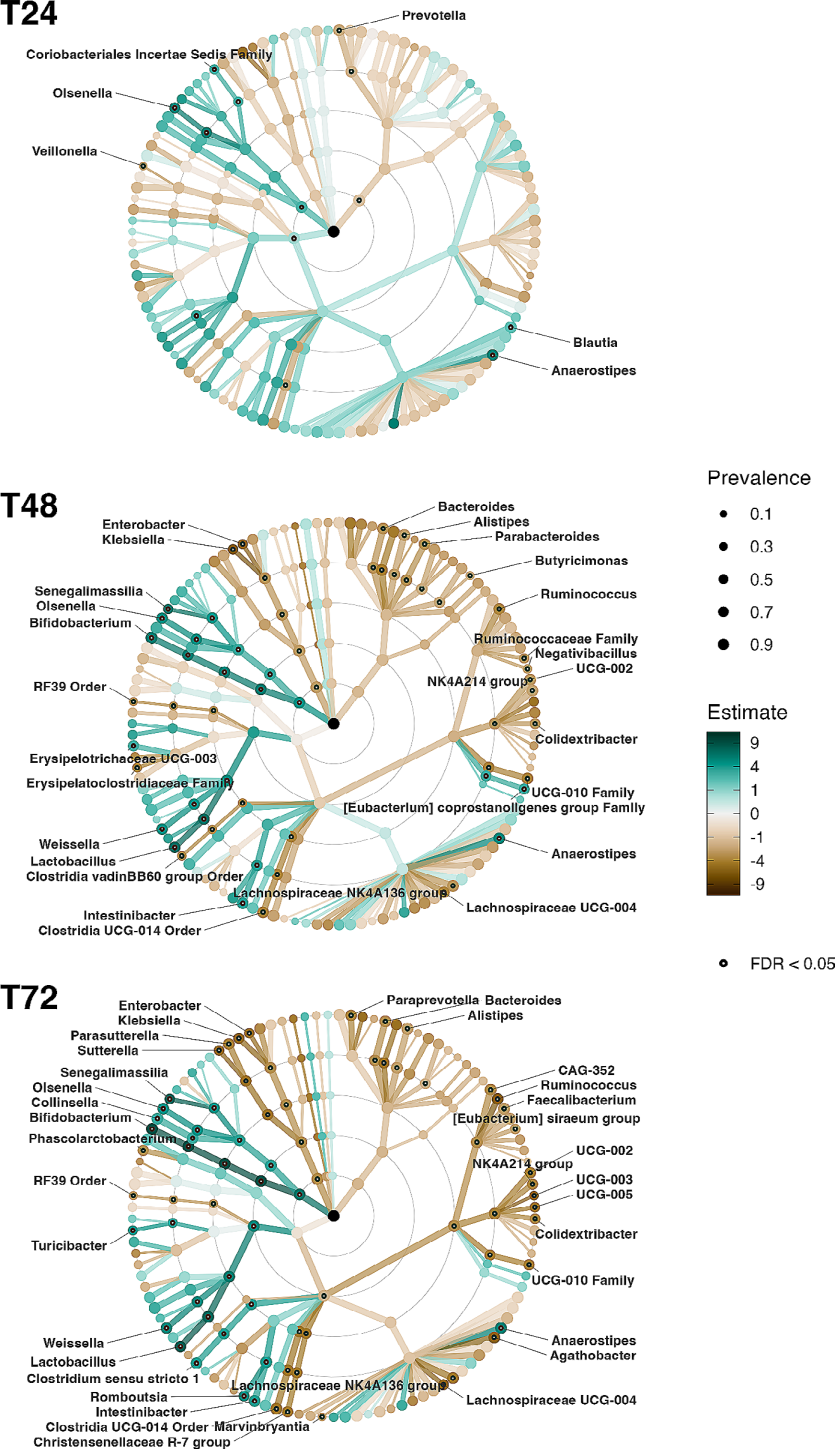
Taxonomic association tree plots indicating genera that showed differential relative abundance between the conditions with prebiotic fibre mixtures (5-FU + M1, 5-FU + M2, 5-FU + M3, 5-FU + M4) and without prebiotic fibre mixtures (5-FU, control), as assessed by linear regression per timepoint. Estimate signifies a point estimate for the regression model coefficient. Higher estimates (brown) refer to enriched taxa abundance in the conditions with prebiotic fibre mixtures, while lower or negative values (green) refer to decreased taxa abundance in the conditions with prebiotic fibre mixtures, as compared to the conditions without

Except for *Tyzzerella*, linear regression confirmed that these genera were differentially abundant between the conditions with and without prebiotic fibre mixtures and indicated an additional set of taxa that showed differential relative abundance between these conditions at T24, T48, or T72, but not at T0 (Fig. 5 and Supplementary Table S4). The first genera that already showed increased abundance due to the addition of prebiotic fibre mixtures at T24 were *Anaerostipes, Blautia*, and *Olsenella*, followed by an increasing number of taxa at T48 and T72 (Fig. 5 and Supplementary Table S4). The stimulating effect on *Blautia* disappeared at later time points (Fig. 5 and Supplementary Figure S6). In addition, relative abundances of *Faecalibacterium* and *CAG_352*, which tended to be increased due to 5-FU, were significantly reduced by prebiotic fibre mixtures at T72, after an initial rise at T24 (Fig. 5 and Supplementary Figure S6). Furthermore, prebiotic fibre mixtures were able to keep the relative abundances of the potentially pathogenic genera *Klebsiella* and *Enterobacter* at relatively low levels, compared to a gradual increase in the conditions without prebiotic fibre mixtures (Fig. 5 and Supplementary Figure S6). On phylum level, Actinomycetota (Actinobacteria) were significantly enriched in the conditions with prebiotic fibre mixtures, while Pseudomonadota (Proteobacteria) were decreased at T48 and T72. At T24 Bacteroidota (Bacteroidetes) were found to be decreased and Bacillota (Firmicutes) were found to be increased (Supplementary Table S4).

We also assessed whether the addition of prebiotic fibre mixtures changed gut microbiota temporal stability. While Bray Curtis distances between T0 and T24 were comparable among all conditions, distances between T0 and T72 appeared to be lower in the 5-FU condition as compared to the control, while distances for the conditions with prebiotic fibre mixtures were slightly higher (Supplementary Figure S8).

Concerning microbial α-diversity, nonparametric marginal models revealed that the Shannon index evolved differently over time depending on whether prebiotic fibre mixtures were applied or not (ATS: *p* = 0.044), which could not be confirmed by repeated measures ANOVA (*p* = 0.230). Observed species richness behaved similarly in runs treated with or without prebiotics fibre mixtures (nonparametric model, ATS: *p* = 0.517, ANOVA: *p* = 0.490, data not shown).

### Effects of 5-FU with and without prebiotic fibre mixtures on cumulative levels of SCFA and BCFA

As a next step, we investigated the effects of 5-FU and the different prebiotic fibre mixtures on cumulative levels of SCFA and BCFA. Average percentages of SCFA and BCFA at the end of the intervention period are given in Table 2. In the 5-FU condition, the acetate : propionate : butyrate ratio was approximately 3 : 1 : 2, while the ratio was approximately 1.5 : 1 : 1.5 in the control condition. In the conditions treated with 5-FU and prebiotic fibre mixtures, the proportion of acetate was higher and the proportion of propionate lower (Table 2). In addition, treatment with prebiotic fibre mixtures increased the proportion of iso-valerate, while it was comparable in 5-FU and control (Table 2).

**Fig. 6:**
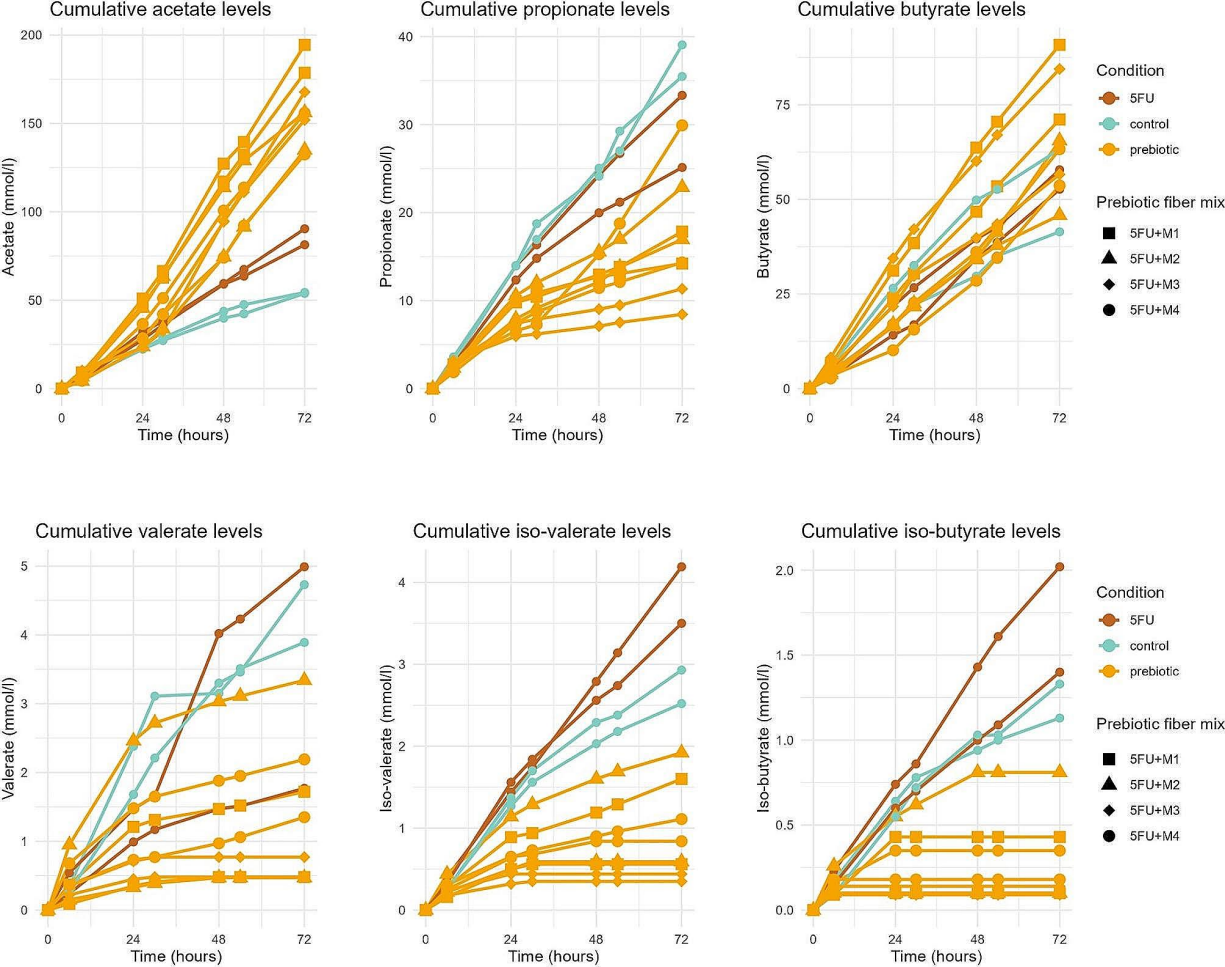
Cumulative levels of SCFA (acetate, propionate, butyrate, and valerate) and BCFA (iso-valerate and iso-butyrate) across different treatments: 5-FU only (5FU), control (no treatment), or 5-FU + prebiotics (prebiotic fibre mixtures)

Concerning SCFA, nonparametric marginal models showed that acetate (ATS: *p* < 0.001), propionate (ATS: *p* < 0.001), and valerate (ATS: *p* = 0.003) evolved differently over time across the three different conditions (control, 5-FU, 5-FU + prebiotics), while butyrate levels were similar (ATS: *p* = 0.523). Except for valerate (*p* = 0.775), these conclusions were confirmed by repeated measures ANOVA (acetate: *p* < 0.001; propionate: *p* < 0.001; butyrate: 0.873). Visualization showed increased levels of acetate in the 5-FU condition and even more increased levels in the 5-FU + prebiotic conditions, while propionate tended to be lower in the 5-FU and 5-FU + prebiotic conditions when compared to the untreated control (Fig. 6). Kruskal Wallis test at T72 did not indicate significant differences between 5-FU, 5-FU + prebiotics, or control at the end of the intervention (Supplementary Table S5). Although butyrate levels seemed to be increased in one M1-run and one M3-run (Fig. 6), this effect was not statistically significant when comparing longitudinal changes between 5-FU, control, and the individual prebiotic fibre mixtures (ATS: *p* = 0.125). According to the nonparametric marginal models, cumulative levels of the BCFA iso-butyrate and iso-valerate behaved significantly different across different treatments (ATS: *p* < 0.001 for both BCFA), which was also confirmed by repeated measures ANOVA (*p* < 0.001 for both BCFA). Visualization indicated increased BCFA levels during 5-FU treatment as compared to control, while levels in the 5-FU + prebiotic conditions were decreased (Fig. 6). Kruskal Wallis test at T72 did not indicate significant differences in cumulative BCFA levels at the end of the intervention (Supplementary Table S5).

**Table 2:** Average percentages of SCFA and BCFA per condition at the end of the intervention (T72)

	5-FU	5-FU + M1	5-FU + M2	5-FU + M3	5-FU + M4	Control
Acetate % of total SCFA	49.4%	65.5%	65.5%	66.4%	63.5%	36.9%
Propionate % of total SCFA	16.8%	5.6%	8.9%	4.1%	9.9%	25.3%
Butyrate % of total SCFA	31.9%	28.5%	24.8%	29.2%	25.8%	35.0%
Valerate % of total SCFA	1.9%	0.4%	0.8%	0.3%	0.8%	2.9%
Iso-butyrate % of total BCFA	30.6%	18.2%	21.9%	22.4%	21.7%	31.1%
Iso-valerate % of total BCFA	69.5%	81.8%	78.1%	77.6%	78.3%	68.9%

## Discussion

To the best of our knowledge, this is the first experimental study using a whole human-derived gut microbial consortium in the TIM-2 model to investigate 5-FU-induced gut microbiota alterations, as well as the potential of prebiotic fibre mixtures to counteract the manifestation of microbial dysbiosis during 5-FU administration. We observed that administration of 5-FU induced changes in gut microbiota composition, but not in overall microbial α-diversity. As a next step, we showed that different prebiotic fibre mixtures increased the abundance of potentially beneficial bacteria (e.g. *Bifidobacterium*, *Lactobacillus, Anaerostipes*) and inhibited the growth of potentially pathogenic bacteria (e.g. *Klebsiella*, *Enterobacter*) in the presence of 5-FU and could therefore be used to prevent 5-FU-induced microbial changes. In addition, we examined the effect of 5-FU with and without prebiotic fibre mixtures on cumulative levels of SCFA and BCFA and observed that acetate increased slightly due to 5-FU, but even more due to 5-FU + prebiotics, while propionate decreased. Compared to the control condition, BCFA were increased due to 5-FU and decreased by 5-FU + prebiotics.

First of all, we were interested in major 5-FU-induced shifts in overall gut microbiota composition. Therefore, we chose to focus on the abundance of the most abundant taxa on phylum and genus level. In contrast to other studies, which were performed in rats [33], mice [23], or with individual bacterial strains [19, 22], we investigated the effects on a whole human-derived microbial consortium. In line with previous results [19, 22, 23, 33], we identified a 5-FU-induced shift in gut microbiota composition. On phylum level, we observed an altered Bacillota/Bacteroidota (Firmicutes/Bacteroidetes) ratio due to 5-FU administration, caused by a relative increase of Bacillota (Firmicutes) and a relative decrease of Bacteroidota (Bacteroidetes). This observation was contradictory to the results from Spanogiannopoulos et al. who reported no significant differences between phyla concerning 5-FU sensitivity [19]. Due to their well-described pro-inflammatory character and association with microbial dysbiosis [56], we expected an increased abundance of Pseudomonadota (Proteobacteria) during 5-FU treatment, which was only found in one of the duplicate experiments. Possibly, Pseudomonadota (Proteobacteria) overgrowth might require a prolonged period of 5-FU administration and/or simulation of inflammation, which should be investigated in future studies.

On genus level, 5-FU seemed to have an inhibitory effect on *Prevotella* and a more stimulating effect on *Bacteroides*, *CAG-352* (a genus from the Ruminococcaceae family), *Faecalibacterium*, and *Blautia.* In line with this, Spanogiannopoulos et al. also found significant growth inhibition of a *Prevotella* strain [19], while *Blautia* strains seemed to be relatively insensitive to 5-FU in different studies [19, 22]. In the same study, the growth of a *Faecalibacterium* strain was also significantly inhibited by 5-FU [19], which was not the case in our experiments. However, it should be noted that sensitivity towards 5-FU was very strain-specific [19, 22], which makes it difficult to compare results on individual strains with our data on genus level in a complex consortium. In contrast to an earlier study, showing reduced α-diversity during 5-FU administration in tumour-bearing mice [23], microbial diversity did not change due to 5-FU in our experiments.

Prebiotic fibres are commonly used in humans to promote a balanced gut microbiota and to counteract microbial dysbiosis. However, limited knowledge exists on whether cancer patients, receiving chemotherapy, would also benefit from prebiotic fibre administration. Therefore, we aimed to examine whether selected prebiotic fibre mixtures also stimulate potentially beneficial bacteria in the presence of 5-FU and could therefore be used to prevent overgrowth of potentially pathogenic bacteria and the manifestation of 5-FU-induced microbial dysbiosis in cancer patients. Our experiments provided several insights which will be of great value for the future design of targeted interventions.

Firstly, all prebiotic fibre mixtures under investigation stimulated *Bifidobacterium*, *Lactobacillus*, and *Anaerostipes*, which was in line with our expectations. While *Bifidobacterium* and *Lactobacillus* are well-known for their beneficial probiotic properties [57], *Anaerostipes* has also been linked to improved host health and production of the SCFA acetate and propionate [58]. On the other hand, it should be noted that the *preTA* operon, involved in 5-FU metabolism, has also been identified in strains belonging to *Anaerostipes* and *Lactobacillus* [19], but it requires further investigation whether these genera actually play an active role in 5-FU metabolism. Besides, the genera *Weissella*, *Olsenella*, and *Senegalimassilia* were associated with prebiotic fibre mixture administration in our experiments. In particular, *Weissella* has been suggested to exert probiotic properties as well as antimicrobial effects against pathogens, such as *E.coli* and *F. nucleatum* [59]. The exact physiological functions of *Olsenella* and *Senegalimassilia*, which both belong to the family of Coriobacteriaceae (which is part of the phylum of Actinomycetota, like *Bifidobacterium*), remain to be investigated.

The initial rise and later decline of the genera *Blautia*, *Faecalibacterium*, and *CAG_352* (belonging to *Clostridium*) are potentially caused by an initial strong effect of 5-FU, which is counteracted by the prebiotic fibre mixtures at later time points. Based on this observation, it might be suggested that clinical interventions with prebiotic fibre mixtures in cancer patients should start before the initiation of 5-FU treatment.

Another interesting finding of our study was that the prebiotic fibre mixtures were able to keep *Klebsiella* and *Enterobacter* at relatively low levels, in contrast to the rising levels in the conditions without prebiotic fibre mixtures. These genera are both members of the Enterobacteriaceae family with pathogenic properties. This bacterial family is of particular interest in the context of 5-FU treatment, since it might be associated with 5-FU efficacy as well as toxicity. As described in the introduction, *E.coli*, also an Enterobacteriaceae family member, has been shown to metabolize 5-FU, thereby decreasing its anti-cancer efficacy [16, 19]. Consequently, it will be an important question for future research whether *Klebsiella* and *Enterobacter* are also involved in 5-FU metabolism and the reduction of treatment efficacy. Furthermore, overgrowth of Enterobacteriaceae is commonly observed in various inflammatory diseases, most likely due to a growth advantage under inflammatory circumstances [60]. As reviewed by Zeng et al., several mechanisms have been proposed to explain the bloom of Enterobacteriaceae in the inflamed gut, which is also thought to further exacerbate inflammation [60]. In the context of anti-cancer treatment, 5-FU-induced dysbiosis might thus be accompanied by a bloom of Enterobacteriaceae, inducing a vicious cycle of increased inflammation, more severe gastrointestinal toxicity, and further Enterobacteriaceae overgrowth. Consequently, the simultaneous stimulation of potentially beneficial bacteria and inhibition of Enterobacteriaceae, as achieved by the prebiotic fibre mixtures under investigation, is considered to represent a promising strategy to counteract the manifestation of 5-FU-induced microbial dysbiosis.

Next to the composition and diversity of the gut microbiota, we also investigated whether 5-FU and prebiotic fibre mixtures had an impact on the production of the bacterial metabolites SCFA and BCFA. SCFA are of special interest in the context of 5-FU-induced toxicity, because they exert various beneficial metabolic, anti-carcinogenic as well as anti-inflammatory effects [29, 61, 62]. BCFA are derived from branched-chain amino acids or ingested via the diet, but their physiological roles are currently not fully understood [30, 63]. They are, however, often considered as a proxy for protein fermentation, which leads to the production of several toxic metabolites, including ammonia, p-cresol, indole, and phenol.

Although prebiotic fibres serve as substrates for SCFA production, previous results concerning which SCFA were stimulated by fibre supplementation were divergent, as reviewed by Vinelli et al. [64]. In our experiments, the administration of prebiotic fibre mixtures in combination with 5-FU considerably increased the production of acetate, which was not surprising because *Lactobacillus* and *Bifidobacterium* are known to be potent acetate producers [30, 65]. Similarly, the decreased propionate levels were in line with the observed changes in taxa abundance, since propionate is mainly produced by the succinate pathway in Bacteroidota (Bacteroidetes), which were found to diminish during our experiments [66]. Next to succinate, lactate is also a precursor of propionate [66]. Therefore, it would be of interest to measure lactate levels in future studies, particularly because *Bifidobacterium* and *Lactobacillus* are also lactate producers [30].

On the other hand, it was surprising that the prebiotic fibre mixtures did not significantly affect butyrate levels, although M1 and M3 seemed to slightly stimulate butyrate production. An increase in butyrate would have been expected based on the fibre selection and because the stimulated *Anaerostipes* is known to be a butyrate producer [66]. However, it should be noted that butyrate can also be produced from acetate and that increased acetate levels could potentially lead to increased butyrate levels if the intervention period would be prolonged [65, 66]. Furthermore, it might be possible that prominent butyrate-producing bacteria (e.g. *Eubacterium*, *Roseburia*, *Coprococcus*) could not grow well under the conditions in the TIM-2 model. In general, differences in SCFA stimulation between studies might also be caused by differences in fibre selection, as well as the dosing and duration of the intervention.

Both BCFA were increased by 5-FU and decreased by prebiotic fibre mixtures, which is of notable interest in view of our recent observation that iso-butyrate was significantly lower in the faeces of CRC patients who showed partial response, compared to patients with stable or progressive disease during capecitabine treatment [67]. Thus, reduction of amino acid degradation and subsequent BCFA production, by administering adequate amounts of prebiotic fibre mixtures, might positively influence tumour response during 5-FU-based treatment and requires further investigation.

This study has its limitations, which arise mainly due to the in vitro design, which mimics but still does not entirely matches a human colon. During the intervention period, 5-FU was injected into the lumen of the model. Consequently, the microbiota were directly exposed to the chemotherapeutic agent, while it reaches the colon more gradually in patients. We took this physiological difference partly into account by choosing a lower dose than the concentration of 5-FU or its metabolites that would be anticipated to be present in vivo (Supplementary Table S1). In this context, it should also be noted that oral capecitabine would be expected to lead to higher chronic colonic exposure, while intravenous 5-FU is expected to induce temporary increases of 5-FU and its metabolites in the colon. In addition, the microbiota used in this study was a pooled microbiota derived from healthy participants. It might be expected that the gut microbiota of cancer patients has a distinct composition and activity. Therefore, ongoing studies also assess the effect of prebiotic fibre mixtures on faecal samples obtained from CRC patients during 5-FU-based chemotherapy. Given the study’s limited statistical power due to only two runs per condition, statistical results should be interpreted cautiously. We have taken this into account as much as possible and used the nparLD R package which can be used to analyse longitudinal measurements in the factorial experiment. Nevertheless our pilot experiments provided valuable insights which need to be validated with larger sample sizes. Because the pH was kept constant in the model, the present experiments do not provide information concerning pH changes upon fibre fermentation, which should be addressed in other settings. An additional limitation is that we cannot exclude that conditions in the model might favour growth of specific gut bacteria, while other bacteria might react more sensitively to this environment, as already discussed in the context of butyrate.

## Conclusions

To conclude, the present experimental study provides pivotal new evidence that targeted modulation of the gut microbiota by prebiotic fibre mixtures might considerably enhance treatment efficacy and reduce the toxicity of 5-FU-based chemotherapy. The use of a whole human-derived microbial consortium in the TIM-2 model closely mimics the human situation and allows conclusions concerning potential clinical benefits. In view of the broad application of 5-FU-based treatments, microbiota modulation by means of prebiotic fibre mixtures might be of considerable benefit to a large number of patients. Therefore, well-designed and controlled clinical studies to investigate the effects, compliance, feasibility, and safety of prebiotic fibre mixture supplementation during 5-FU-based chemotherapy are urgently required.

### Electronic supplementary material

Below is the link to the electronic supplementary material.

Supplementary Material 1

## Data Availability

Raw sequencing data were submitted to Qiita [68] and deposited in the European Nucleotide Archive (ENA). Data are accessible via project PRJEB73821 or the secondary study accession code ERP158560. Additional data used and/or analysed are available from the corresponding author on reasonable request.

## Abbreviations

5-FU: 5-Fluorouracil
ASVs: Amplicon Sequence Variants
ATS: ANOVA-type statistic
BCFA: Branched-chain fatty acids
CLR: Centered log-ratio
CRC: Colorectal cancer
ENA: European Nucleotide Archive
FDR: False Discovery Rate
FdUMP: Fluorodeoxyuridine monophosphate
FdUTP: Fluorodeoxyuridine triphosphate
FMT: Faecal microbiota transplantation
FUTP: Fluorouridine triphosphate
iFOBT: Immunochromatographic faecal occult blood test
lcFOS: Long-chain fructo-oligosaccharides (lcFOS)
lyPect: Low viscosity pectin
MUMC+: Maastricht University Medical Center
PBS: Phosphate-buffered saline
PCA: Principal Component Analysis
SCFA: Short-chain fatty acids
scGOS: Short-chain galacto-oligosaccharides
SIEM: Standard ileal efflux medium
TIM-2: TNO in vitro model of the colon
