# Supplementary material for: Prebiotic fibre mixtures counteract the manifestation of gut microbial dysbiosis induced by the chemotherapeutic 5-Fluorouracil (5-FU) in a validated in vitro model of the colon
Source: BMC Microbiol. 2024 Jun 26;24:222. doi: 10.1186/s12866-024-03384-4 (PMC11200995; doi:10.1186/s12866-024-03384-4)
Supplement: Supplementary file 1 — Supplementary Material 1 [file 12866_2024_3384_MOESM1_ESM.pdf]

Supplementary Tables and Figures

Table S1: *Calculation of the 5-FU dose for* in vitro *experiments*

Fecal excretion of capecitabine or its metabolites (1)	2.64%
Average dose capecitabine applied in our study population in 24h	4000mg
Dose expected to reach the colon in 24h	2.64% x 4000mg = 105.6mg
Dose per timepoint if administered 2x daily	105.6mg / 2 = 52.8mg
50% of dose per timepoint	52.8mg /2 = 26.4mg

Table S2: *Amplicon primers used for the sequencing of the V3-V4 regions of the 16S rRNA gene*

16S Amplicon PCR Forward Primer = 5' TCGTCGGCAGCGTCAGATGTGTATAAGAGACAGCCTACGGGNGGCWGCAG
16S Amplicon PCR Reverse Primer = 5' GTCTCGTGGGCTCGGAGATGTGTATAAGAGACAGGACTACHVGGGTATCTAATCC

Table S3: *Clinical characteristics of the feces donors*

**Clinical characteristics**	
**Age – Years**	**mean (SD)**<br>64.8 (6.3)
**BMI - kg/m²**	**mean (SD)**<br>22.43 (2.57)
**Current smoking**	**n**
Yes	3
No	6
Missing	1
**Past smoking**	**n**
**Yes**	4
**No**	5
**Missing**	1
**Use of Medication**	**n**
Yes	6
No	4
**Type of Medication***	**n**
Angiotensin receptor blockers	1
Anti-viral agents	1
β2-adrenergic receptor agonists	1
Calciumantagonists	1
Corticosteroids	1
Diuretics	1
Prostaglandin f-analog with β-adrenergic antagonist	1
Serotonin reuptake inhibitors	2
Statins	1
Triptans	1
Vitamine or mineral supplements	4

* use of more than 1 medication per individual leads to a sum >10.

Table S4: *Bacterial taxa on phylum and genus level which were found to be differentially abundant between prebiotic (5-FU+M1, 5-FU+M2, 5-FU+M3, 5-FU+M4) and non-prebiotic (5-FU, control) conditions, according to linear regression.*

**T0**					
**Taxon**	**Rank**	**estimate**	**std.error**	**statistic**	**p.adjusted**
No significant taxa					
**T24**					
**Taxon**	**Rank**	**estimate**	**std.error**	**statistic**	**p.adjusted**
Bacteroidota	Phylum	-1.046	0.366	-2.859	0.040
Firmicutes	Phylum	0.699	0.216	3.241	0.040
Actinobacteriota	Phylum	2.594	0.862	3.011	0.040
Anaerostipes	Genus	5.729	0.834	6.868	0.005
Blautia	Genus	1.941	0.382	5.081	0.025
f__Coriobacteriales_Incertae_Sedis Family	Genus	2.945	0.606	4.860	0.025
Prevotella	Genus	-1.973	0.442	-4.466	0.027
Olsenella	Genus	5.838	1.267	4.607	0.027
Veillonella	Genus	-2.412	0.569	-4.240	0.033
**T48**					
**Taxon**	**Rank**	**estimate**	**std.error**	**statistic**	**p.adjusted**
Proteobacteria	Phylum	-2.567	0.598	-4.294	0.011
Actinobacteriota	Phylum	4.709	1.524	3.091	0.040
Ruminococcus	Genus	-3.634	0.667	-5.452	0.016
NK4A214_group	Genus	-2.225	0.403	-5.525	0.016
Anaerostipes	Genus	5.363	1.191	4.502	0.027
UCG-002	Genus	-2.645	0.618	-4.280	0.027
f__Erysipelatoclostridiaceae Family	Genus	-3.912	0.891	-4.388	0.027
f__UCG-010 Family	Genus	-3.884	0.909	-4.273	0.027
Enterobacter	Genus	-5.615	1.210	-4.642	0.027
Klebsiella	Genus	-6.495	1.650	-3.935	0.035
Olsenella	Genus	4.983	1.307	3.812	0.035
Senegalimassilia	Genus	6.750	1.685	4.007	0.035
f__Ruminococcaceae Family	Genus	-2.780	0.729	-3.815	0.035
o__Clostridia_vadinBB60_group Order	Genus	-3.424	0.913	-3.749	0.035
Negativibacillus	Genus	-2.447	0.659	-3.714	0.035
Alistipes	Genus	-2.427	0.676	-3.590	0.040
Bifidobacterium	Genus	6.107	1.793	3.406	0.041
Bacteroides	Genus	-2.978	0.892	-3.341	0.041
Lactobacillus	Genus	7.085	2.155	3.288	0.041
Lachnospiraceae_NK4A136_group	Genus	-2.829	0.831	-3.405	0.041
o__Clostridia_UCG-014 Order	Genus	-3.896	1.151	-3.384	0.041
Lachnospiraceae_UCG-004	Genus	-3.709	1.096	-3.384	0.041
Weissella	Genus	4.815	1.462	3.294	0.041
Colidextribacter	Genus	-2.771	0.843	-3.287	0.041
Erysipelotrichaceae_UCG-003	Genus	2.807	0.801	3.505	0.041
Butyricimonas	Genus	-2.065	0.638	-3.237	0.042
f__[Eubacterium]_coprostanoligenes_group Family	Genus	3.155	0.984	3.207	0.043
o__RF39 Order	Genus	-3.135	0.997	-3.144	0.046
Parabacteroides	Genus	-3.111	1.001	-3.108	0.047
Intestinibacter	Genus	3.752	1.218	3.081	0.047
**T72**					
**Taxon**	**Rank**	**estimate**	**Std.error**	**statistic**	**p.adjusted**
Actinobacteriota	Phylum	6.681	0.407	16.398	<0.001
Proteobacteria	Phylum	-3.355	0.598	-5.611	<0.001
Bifidobacterium	Genus	9.317	0.412	22.621	<0.001
Senegalimassilia	Genus	7.989	0.459	17.397	<0.001
o__Clostridia_UCG-014 Order	Genus	-4.596	0.651	-7.062	0.002
Lactobacillus	Genus	8.371	1.299	6.444	0.002
Anaerostipes	Genus	5.872	0.912	6.439	0.002
Agathobacter	Genus	-5.808	0.913	-6.359	0.002

UCG-003	Genus	-5.694	0.972	-5.861	0.003
Romboutsia	Genus	5.192	0.888	5.848	0.003
f__UCG-010 Family	Genus	-4.075	0.725	-5.619	0.004
Weissella	Genus	5.552	1.046	5.307	0.005
Collinsella	Genus	3.176	0.625	5.086	0.005
Sutterella	Genus	-5.456	1.080	-5.051	0.005
Lachnospiraceae_UCG-004	Genus	-5.591	1.095	-5.107	0.005
Parasutterella	Genus	-3.924	0.751	-5.222	0.005
Intestinibacter	Genus	3.857	0.793	4.862	0.006
UCG-005	Genus	-4.582	0.989	-4.633	0.008
Faecalibacterium	Genus	-2.548	0.570	-4.472	0.009
Lachnospiraceae_NK4A136_group	Genus	-4.507	1.015	-4.442	0.009
Clostridium_sensu_stricto_1	Genus	3.924	0.874	4.491	0.009
Ruminococcus	Genus	-6.245	1.436	-4.349	0.010
Alistipes	Genus	-2.452	0.602	-4.071	0.015
NK4A214_group	Genus	-3.793	0.943	-4.024	0.015
Turicibacter	Genus	4.461	1.128	3.954	0.016
Enterobacter	Genus	-4.981	1.345	-3.704	0.022
Phascolarctobacterium	Genus	-3.123	0.854	-3.658	0.023
Olsenella	Genus	4.793	1.331	3.602	0.024
Bacteroides	Genus	-3.133	0.889	-3.524	0.026
UCG-002	Genus	-4.071	1.197	-3.401	0.031
CAG-352	Genus	-3.382	1.068	-3.166	0.041
Klebsiella	Genus	-5.378	1.713	-3.140	0.041
[Eubacterium]_siraeum_group	Genus	-2.431	0.767	-3.169	0.041
Colidextribacter	Genus	-3.564	1.134	-3.143	0.041
Christensenellaceae_R-7_group	Genus	-4.378	1.422	-3.078	0.042
Marvinbryantia	Genus	-2.628	0.851	-3.089	0.042
o__RF39 Order	Genus	-2.757	0.901	-3.061	0.042
Paraprevotella	Genus	-3.755	1.253	-2.998	0.045

Table S5: Results from Kruskal Wallis Test at T72 to test for cross-sectional differences in cumulative SCFA/BCFA concentrations between 5-FU, 5-FU+prebiotics and control at T72

**Kruskal Wallis Test**	**X^2**	**df**	**p**
**Acetate**	9.846	5	0.080
**Propionate**	9.308	5	0.097
**Butyrate**	5.231	5	0.388
**Valerate**	6.619	5	0.251
**Iso-butyrate**	8.588	5	0.127
**Iso-valerate**	9.615	5	0.087

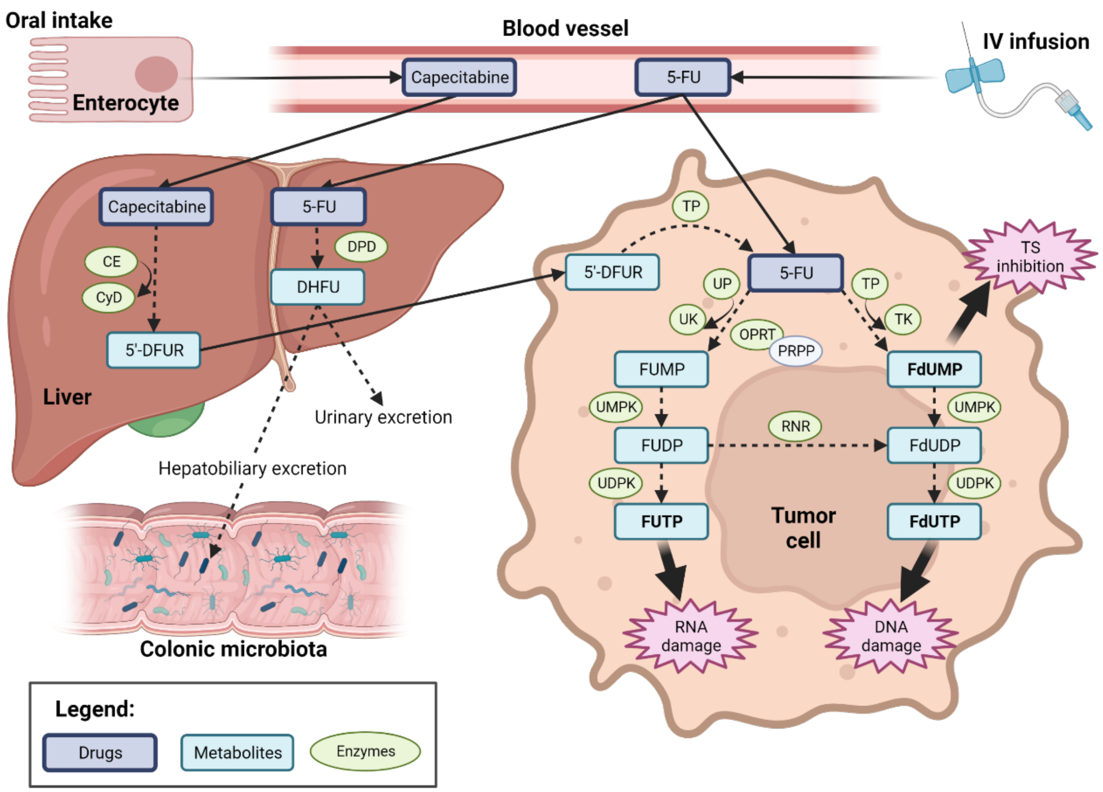

Figure S1: 5-fluorouracil (5-FU) and capecitabine metabolism, excretion and antineoplastic effects. *Based on* (1-10), *created with BioRender.com. 5-FU is administered intravenously and relies on intracellular activation to yield three active metabolites:* ***1****- fluorodeoxyuridine monophosphate (FdUMP), which inhibits thymidylate synthase (TS), leading to an imbalance of deoxynucleotides and increased dUMP levels, thereby impairing DNA synthesis and repair and causing DNA damage;* ***2****- fluorouridine triphosphate (FUTP), which is incorporated into RNA, disrupting RNA processing and function; and* ***3****- fluorodeoxyuridine triphosphate (FdUTP) which causes DNA damage by incorporation into DNA.*

In more detail, 5-FU is converted into FdUMP through subsequent activity of thymidine phosphorylase (TP) and thymidine kinase (TK). Alternatively, 5-FU can be converted into fluorouridine monophosphate (FUMP) directly by orotate phosphoribosyl transferase (OPRT), in the presence of phosphoribosyl pyrophosphate (PRPP), or indirectly by uridine phosphorylase (UP) and uridine kinase (UK) activity. FUMP is then further metabolized into fluorouridine diphosphate (FUDP) by uridine monophosphate kinase (UMPK). FUDP is either phosphorylated into FUTP by uridine diphosphate kinase (UDPK) or converted into fluorodeoxyuridine diphosphate (FdUDP) by ribonucleotide reductase (RNR). FdUDP can then be further phosphorylated into FdUTP or reduced into FdUMP. The majority of 5-FU however is rapidly catabolized into inactive metabolites by DPD, which is mainly expressed in the liver. These metabolites are excreted mainly renally or hepatobiliary.

Capecitabine is orally administered and rapidly absorbed, before it is metabolized into 5-FU. First it is converted into 5'-deoxy-5-fluorouridine (5'-DFUR) by carboxylesterase (CE) and cytidine deaminase (CyD), primarily in the liver. TP is responsible for the final conversion of 5'-DFUR into 5-FU. TP is present at higher concentrations in tumor tissue.

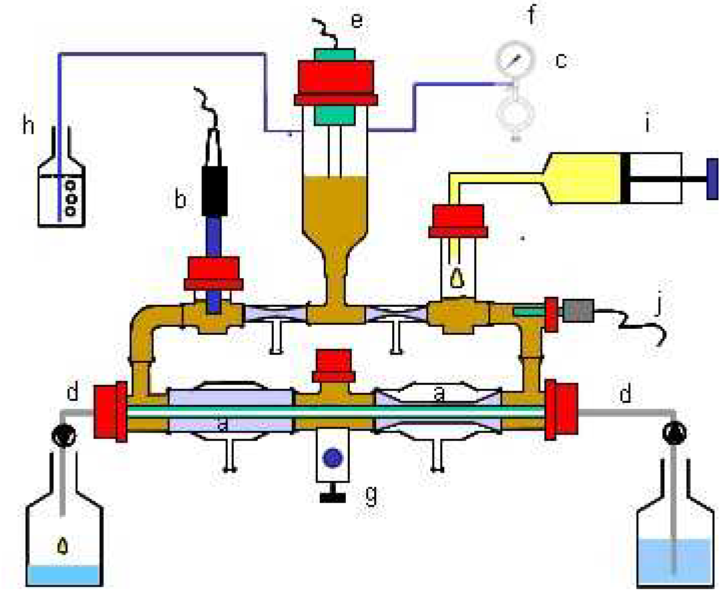

Figure S2: *Schematic representation of TIM-2. a: peristaltic compartments; b: pH-electrode; c: alkali pump; d: dialysis liquid circuit with hollow fibres; e: level-sensor; f: N2 gas inlet; g: sampling-port; h: gas outlet; i: 'ileal delivery' container; j: temperature sensor.*

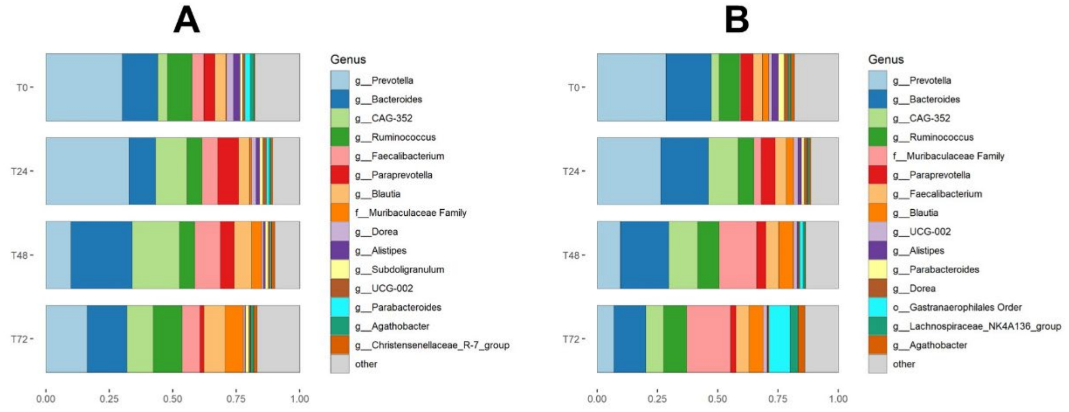

Figure S3: *Changes in relative abundance of the most common genera which were induced by 2x52.8mg 5-FU per day (A) or by 2x26.4mg 5-FU per day (B). Both doses led to comparable shifts in taxa abundance.*

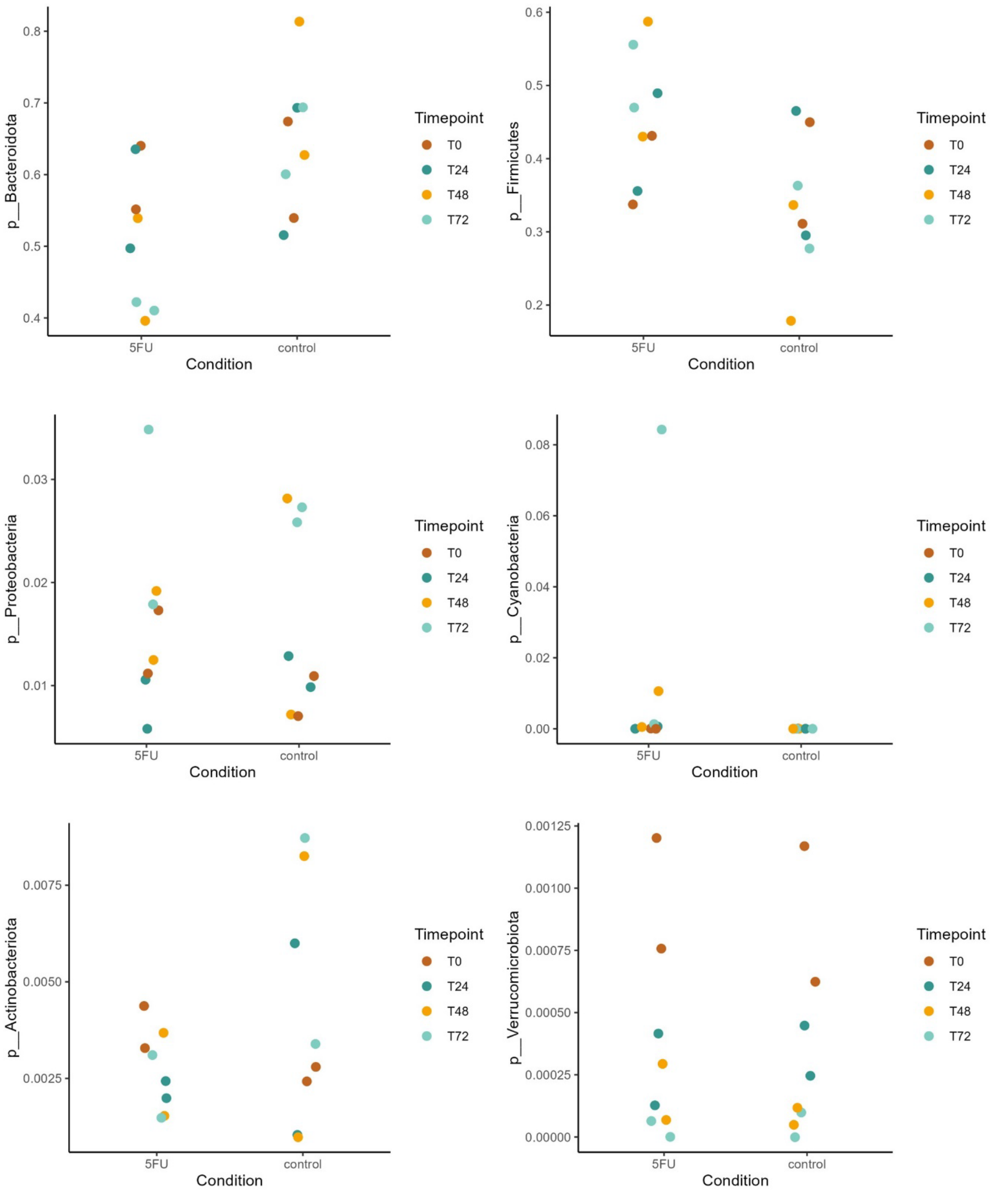

Figure S4: *Relative abundances of the most common phyla during the intervention period in 5-FU compared to control in the individual runs.*

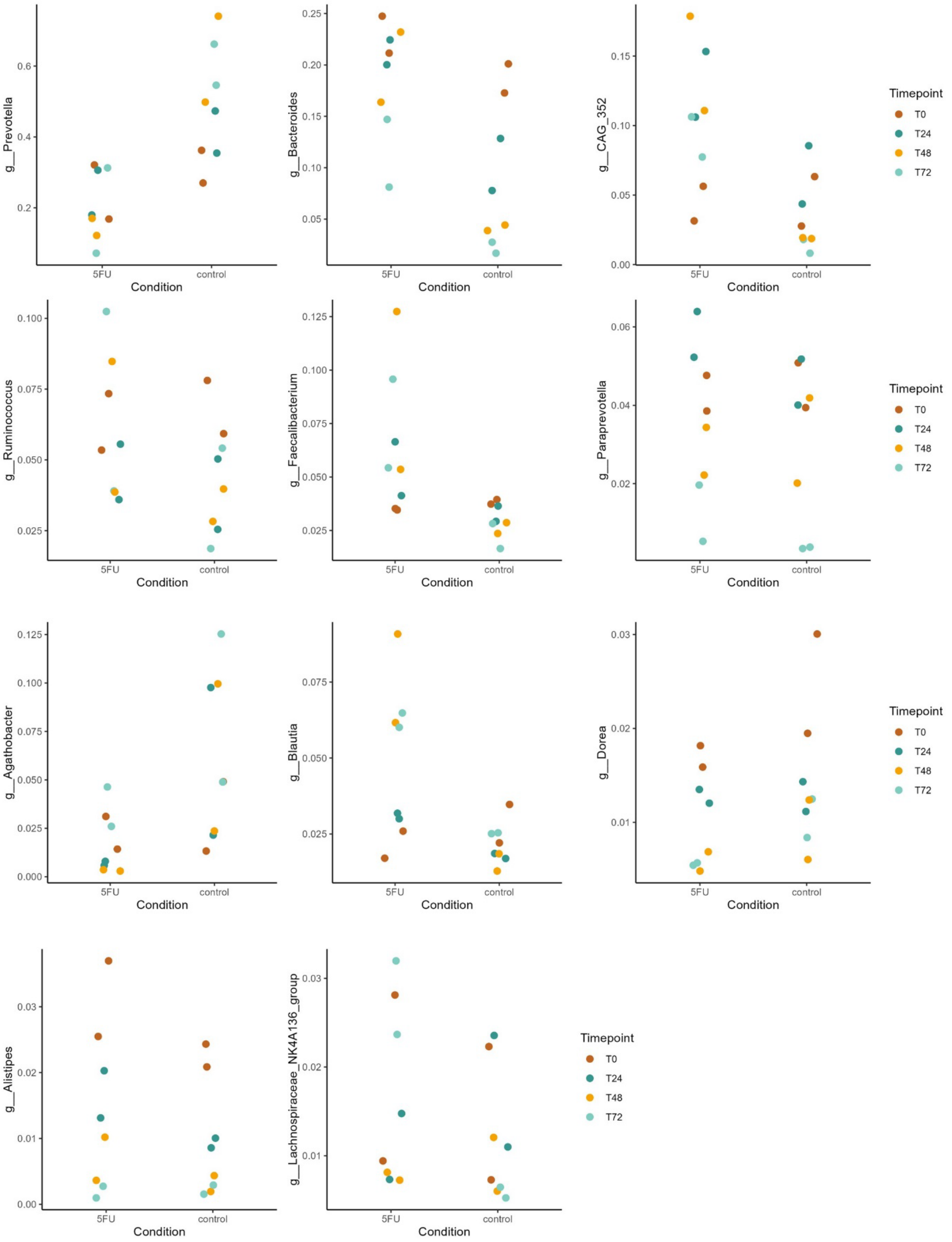

Figure S5: *Relative abundances of the most common genera during the intervention period in 5-FU compared to control in the individual runs.*

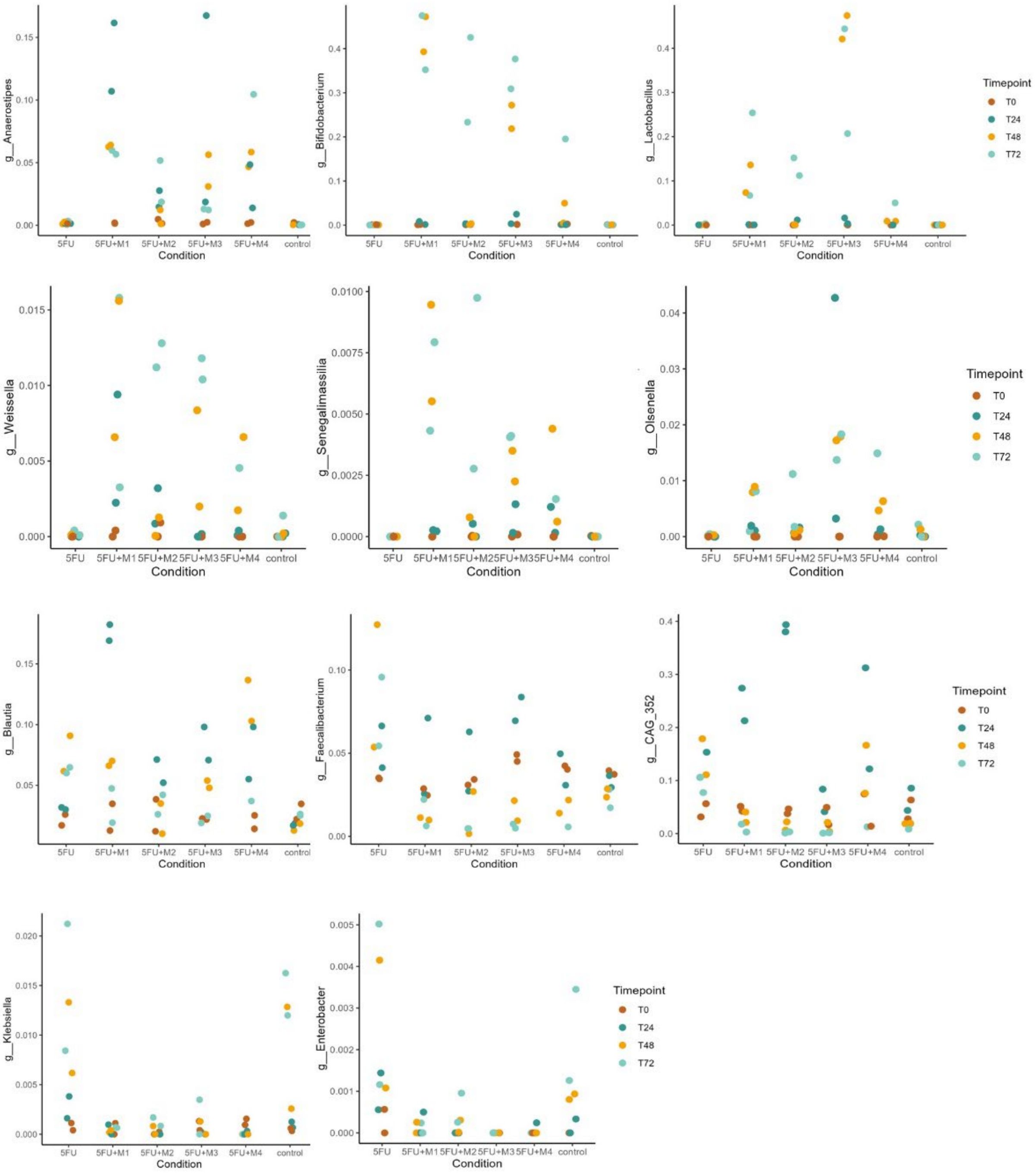

Figure S6: *Relative abundances of genera of interest during the intervention period in the individual runs. Genera of interest were identified based on composition plots, PCA and taxonomic association tree plots.*

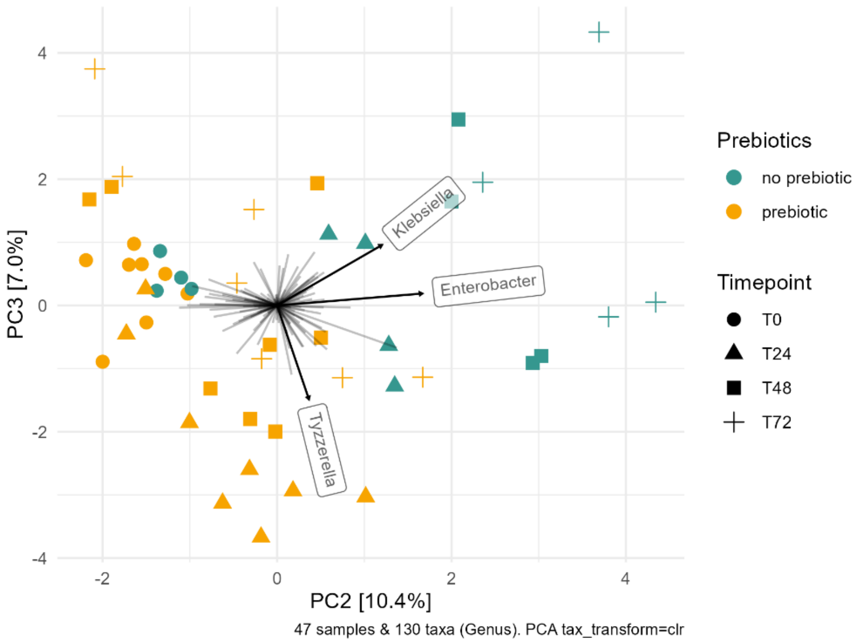

Figure S7: *Ordination plot (PC2 and PC3) derived from unconstrained PCA based on clr transformed data, showing differences in longitudinal microbiota shifts between prebiotic (5-FU+M1, 5-FU+M2, 5-FU+M3, 5-FU+M4) and non-prebiotic (5-FU, control) conditions. Taxa which were present in less than 3 of the 47 samples (min_prevalence=0.05) were filtered out. Vectors indicate the top three genera which contributed most to the observed variation between prebiotic and non-prebiotic conditions along PC2.*

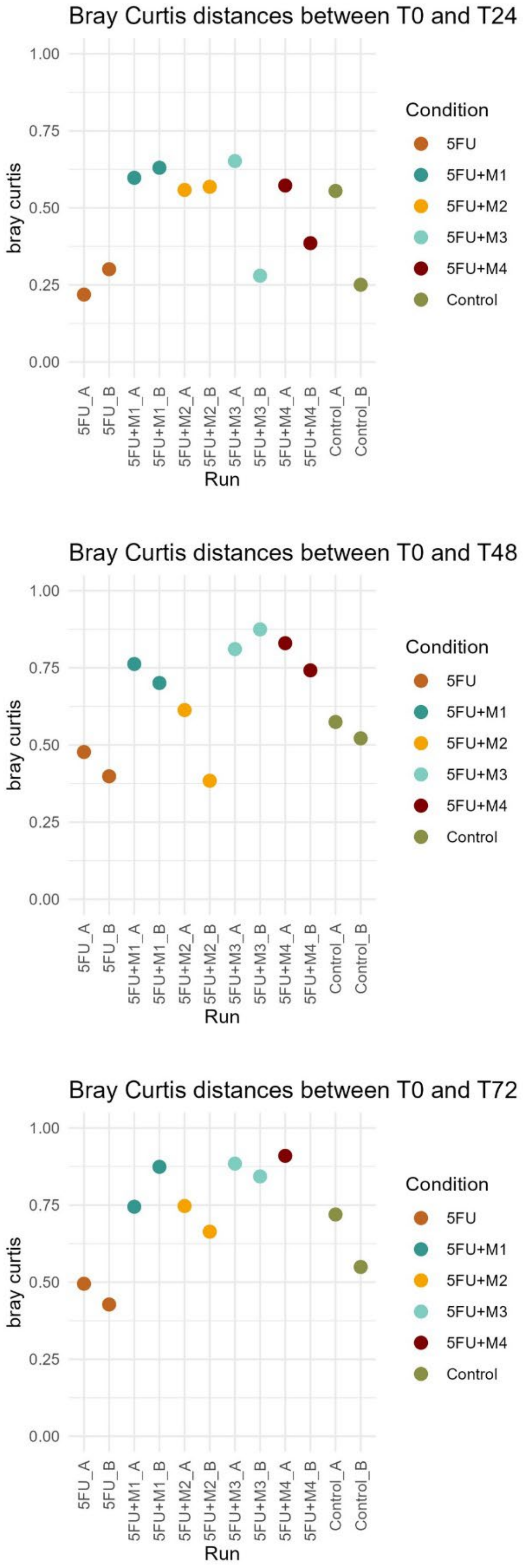

Figure S8: *Temporal (in)stability in microbial communicty structure (β-diversity). Bray-Curtis distances between baseline (T0) and T24, T48 and T72 respectively, among different conditions for individual runs.*

References

1. Reigner B, Blesch K, Weidekamm E. Clinical pharmacokinetics of capecitabine. Clin Pharmacokinet. 2001;40(2):85-104.
2. Alvarez P, Marchal JA, Boulaiz H, Carrillo E, Velez C, Rodriguez-Serrano F, et al. 5-Fluorouracil derivatives: a patent review. Expert Opin Ther Pat. 2012;22(2):107-23.
3. Longley DB, Harkin DP, Johnston PG. 5-fluorouracil: mechanisms of action and clinical strategies. Nature reviews Cancer. 2003;3(5):330-8.
4. Diasio RB, Harris BE. Clinical pharmacology of 5-fluorouracil. Clin Pharmacokinet. 1989;16(4):215-37.
5. Milano G, McLeod HL. Can dihydropyrimidine dehydrogenase impact 5-fluorouracil-based treatment? Eur J Cancer. 2000;36(1):37-42.
6. Miura K, Kinouchi M, Ishida K, Fujibuchi W, Naitoh T, Ogawa H, et al. 5-fu metabolism in cancer and orally-administrable 5-fu drugs. Cancers (Basel). 2010;2(3):1717-30.
7. Tomao F, Caruso G, Musacchio L, Di Donato V, Petrella MC, Verrico M, et al. Capecitabine in treating patients with advanced, persistent, or recurrent cervical cancer: an active and safe option? Expert Opin Drug Saf. 2021;20(6):641-50.
8. Johnston PG, Kaye S. Capecitabine: a novel agent for the treatment of solid tumors. Anticancer Drugs. 2001;12(8):639-46.
9. Miwa M, Ura M, Nishida M, Sawada N, Ishikawa T, Mori K, et al. Design of a novel oral fluoropyrimidine carbamate, capecitabine, which generates 5-fluorouracil selectively in tumours by enzymes concentrated in human liver and cancer tissue. Eur J Cancer. 1998;34(8):1274-81.
10. Schüller J, Cassidy J, Dumont E, Roos B, Durston S, Banken L, et al. Preferential activation of capecitabine in tumor following oral administration to colorectal cancer patients. Cancer Chemother Pharmacol. 2000;45(4):291-7.
